# Sophorolipid: a glycolipid biosurfactant as a potential therapeutic agent against COVID-19

**DOI:** 10.1080/21655979.2021.1997261

**Published:** 2021-12-14

**Authors:** Amita Daverey, Kasturi Dutta, Sanket Joshi, Achlesh Daverey

**Affiliations:** a Department of Neurosurgery, University of Nebraska Medical Center, Omaha, NE, USA; b Department of Biotechnology and Medical Engineering, National Institute of Technology, Rourkela, India; c Oil & Gas Research Center, Central Analytical and Applied Research Unit, Sultan Qaboos University, Muscat, Oman; d School of Environment and Natural Resources, Doon University, Dehradun, India; e School of Biological Sciences, Doon University, Dehradun, India

**Keywords:** Antiviral, biosurfactant, COVID-19, glycolipids, sophorolipids, SARS-COV-2

## Abstract

Biosurfactants are natural surfactants produced by a variety of microorganisms. In recent years, biosurfactants have garnered a lot of interest due to their biomedical and pharmaceutical applications. Sophorolipids are glycolipid types of biosurfactants produced by selected nonpathogenic yeasts. In addition to the detergent activity (reduction in surface and interfacial tension), which is commonly utilized by biomedical applications, sophorolipids have shown some unique properties such as, antiviral activity against enveloped viruses, immunomodulation, and anticancer activity. Considering their antiviral activity, the potential of sophorolipids as an antiviral therapy for the treatment of COVID-19 is discussed in this review. Being a surfactant molecule, sophorolipid could solubilize the lipid envelope of SARS-CoV-2 and inactivate it. As an immunomodulator, sophorolipid could attenuate the cytokine storm caused by the SARS-CoV-2 upon infection, and inhibit the progression of COVID-19 in patients. Sophorolipids could also be used as an effective treatment strategy for COVID-19 patients suffering from cancer. However, there is limited research on the use of sophorolipid as a therapeutic agent for the treatment of cancer and viral diseases, and to modulate the immune response. Nevertheless, the multitasking capabilities of sophorolipids make them potential therapeutic candidates for the bench-to-bedside research for the treatment of COVID-19.

## 1. COVID-19 and SARS-CoV-2

The world is witnessing the global coronavirus disease 2019 (COVID-19) pandemic that killed more than 4,265,903 people worldwide (>200,000,000 confirmed cases of COVID-19) as of 6 August 2021, and the numbers are still counting [1]. The disease is caused by a novel coronavirus, SARS-CoV-2 (Severe Acute Respiratory Syndrome Coronavirus 2), which is highly pathogenic, and has the same zoonotic origin as SARS-CoV (Severe Acute Respiratory Syndrome Coronavirus) and MERS-CoV (Middle East respiratory syndrome coronavirus) that caused outbreaks in 2002–2003, and 2012 and 2015, respectively [2]. SARS-CoV-2 causes severe respiratory illnesses, such as acute respiratory distress syndrome (ARDS) and pneumonia [3]. In addition, severe cases showed hepatic, gastrointestinal, and neurological complications that could lead to mortality [4]. The transmission of COVID-19 is reported to be human-to-human through respiratory droplets, direct contact with the infected patients, or touching infected surfaces [5,6]. Airborne transmission of COVID-19, particularly in indoor has also been in debate [7,8].

Though several vaccines are available now in the market, they have their own merits and limitations. To date, there is no proven effective treatment for COVID-19, therefore, prevention measures such as personal hygiene, social distancing, and isolation are the most effective way to minimize the transmission of a virus. Several clinical trials of possible treatments based on anti-viral, vaccines, anti-inflammatory, anticoagulants are in progress [Ahn et al., 2020, 9, 10]. The main treatments being used are drug therapy (use of remdesivir, lopinavir/ritonavir combination, ivermectin, nitazoxanide, and chloroquine/hydroxychloroquine), passive immunization (by transferring ‘convalescent sera’ from a person who recovered from COVID-19), and respiratory therapy [11-13]. Some of them remain controversial to use for the treatment of COVID-19. For example, WHO doesn’t recommend the use of hydroxychloroquine for the treatment of COVID-19, while the use of ivermectin to treat COVID-19 is only advised within clinical trials [14,15]. Antiviral therapies currently used are based on the experience gained during SARS-CoV and MERS-CoV epidemics [9,16,17]. Researchers are also exploring different targets for the potential drugs/vaccines to kill the SARS-CoV2. One of the alternative strategies could be targeting the structural genes for the ‘S’ protein, or envelope, or membrane protein with small interfering RNAs [4]. The lipid membrane of SARS-CoV-2 has also been suggested as a possible target to kill the virus [18].

Recent perspectives and research studies [19–22] has described the possible role of biosurfactants in COVID-19 management as antiviral, anti-inflammatory, disinfecting agents, cleaning and eco-friendly hand-washing agents. It is hypothesized that biosurfactants, which are microbial-derived surfactants could attack the lipid membrane of the enveloped SARS-CoV-2. In this mini-review, we explain why sophorolipids (SLs) – glycolipid type of biosurfactants, could be a potential therapeutic intervention in managing COVID-19. The hypothetical mechanism of action of SLs in killing the SARS-CoV-2 and inhibiting the progression of COVID-19 is also proposed.

## 2. Biosurfactants and COVID-19

Biosurfactants are surface-active amphiphilic (having both hydrophilic and lipophilic groups in a single moiety) biomolecules produced by microorganisms (bacteria, fungi, and yeasts), as extracellular secondary metabolites [22–24]. Structurally, they are highly diverse and can be classified as glycolipids, lipopeptides, phospholipids, lipopolysaccharides, fatty acids, and polymers [23,24]. They find applications in cosmetics, personal care products, and household cleanings, while their potential in pharmaceuticals, environmental clean-up, agriculture, and food industries are also being evaluated by the researchers [24–27]. Being surfactant molecules they can solubilize the membrane, and they have been reported as antiviral agents, particularly for enveloped viruses including coronaviruses [28, Johnson et al., 2019; 20, 29]. Table 1 compiles the biosurfactants reported to have antiviral activities against various enveloped viruses. It can be seen from Table 1, the biosurfactants having antiviral activities belong to glycolipids and lipopeptide classes. The suggested mechanism of antiviral activity of the biosurfactants is the disruption of the viral lipid membrane [Vollenbroich et al., 1997].

**Table 1.** Biosurfactants having antiviral properties

Chemical Class	Biosurfactant	Microbial Source	Activity against viruses	References
Glycoplipids	Rhamnolipids	*Pseudomonas aeruginosa* 196; *Pseudomonas sp.*	Herpesvirus; Tobacco mosaic virus [Crop viral infection); Bovine coronavirus; SARS-CoV-2	20, 71,72
	Sophorolipids	*Candida bombicola [Starmerella bombicola*]	Human HIV, Epstein-Barr virus, and Influenza virus	45, 47, 48
Lipopeptide	Surfactin	*Bacillus subtilis*	Coronavirus (human CoV-229E, MERS-CoV or SARS-CoV]; influenza A virus [IAV); Herpes simplex virus; Simian immunodeficiency virus	Johnson et al., 2019 Vollenbroich et al., 1997
	Fengycin	*Bacillus amyloliquefaciens*	Cucumber mosaic virus	29
	Surfactin or Fengycin	*Bacillus subtilis* fmbj	Pseudorabies Virus, Porcine Parvovirus, Newcastle Disease Virus and Infectious Bursal Disease Virus	28

Recently, biosurfactants due to their detergent, pharmaceutical, and antiviral activities have been suggested to play a critical role in managing the current COVID-19 pandemic. Moreover, the mechanism of anti-inflammatory properties of biosurfactants including SLs was reviewed by [22]. The authors highlighted the potential role of anti-inflammatory properties of the biosurfactants in managing the COVID-19 pathogenesis. In another study, rhamnolipids (RLs) have been successfully tested as disinfectants against the enveloped viruses, including the bovine coronavirus [20]. The authors also overserved antiviral activity of the RLs-coated surfaces (plastic and fabric) and therefore, suggested that the RLs-coated mask and plastic surfaces can be used to stop the spread of the COVID-19 virus. In another study conducted by [30], cotton fabrics coated with lactonic sophorolipids (SLs) along with 1,2,3,4- butanetetracarboxyic acid showed antimicrobial activity against *Staphylococcus aureus*. Because SLs and other biosurfactants have antiviral activities against several enveloped viruses, they should also show similar antiviral activities against coronaviruses. Peptidoglycan-associated surfactin biosurfactant produced from *Bacillus subtilis* has been found as a virucidal agent against enveloped viruses including SARS-CoV (Johnson et al., 2019). These studies suggest that biosurfactants can play potential roles in the management of COVID-19 in multiple ways. However, so far, a specific biosurfactant has not been studied or proposed as a therapeutic agent against SARS-CoV-2 or COVID-19 treatment.

## 3. Sophorolipids – an introduction

**Figure 1.**
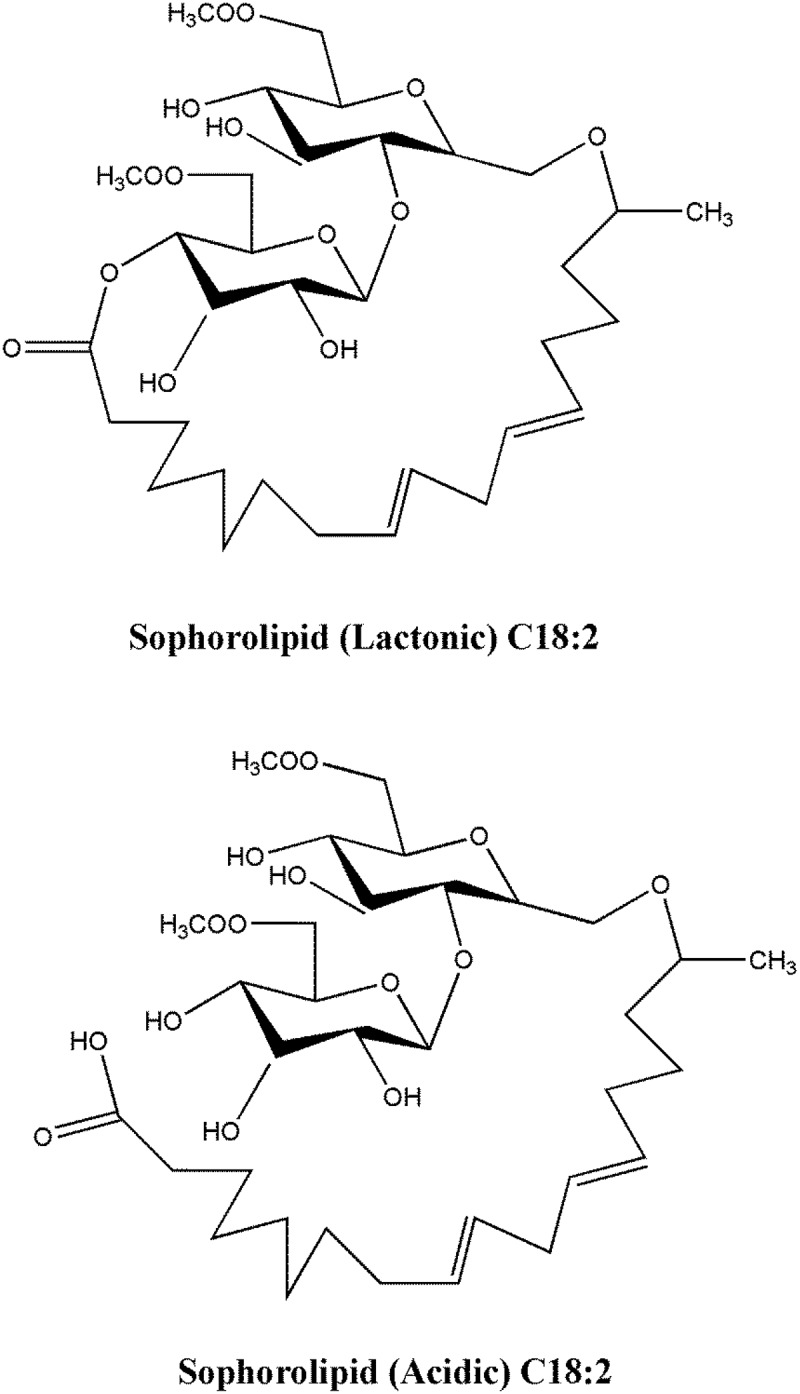
Structure of lactonic and acidic forms of sophorolipids

SLs are a group of natural surfactants produced by selected nonpathogenic yeasts (*Starmerella bombicola a teleomorph of Candida bombicola, C. apicola, C. bogoriensis, etc*.) [31–34]. SLs belong to the glycolipids class of biosurfactants, which are low molecular weight extracellular metabolites. Structurally, it is an amphiphilic molecule that consists of a sophorose sugar (hydrophilic group), which is glycosidically linked to a hydroxylated fatty acid chain (hydrophobic). The fatty acid chain has variable carbon atoms with a different degree of saturation, which depends on the microbial strain and hydrophobic carbon source used for its production in the medium [35]. It can be synthesized in the laboratory as a mixture of lactonic and acidic SLs [31,32]. Examples of lactonic and acidic SLs are shown in Figure 1.

Being surfactant molecules, SLs are highly efficient in reducing the surface and interfacial tensions between two phases. For example, the minimum surface tension achieved by the addition of a mixture of SLs in water is 34.18–28.56 mN/m, and the interfacial tension against *n*-hexane (a nonpolar solvent) is 0.99 mN/m [31,32]. The critical micelle concentration of a mixture of SLs produced by *Candida bombicola* using a mixed hydrophilic carbon source (whey and glucose) and oleic acid was found to be 27.17 mg/L [31]. These properties suggest that SLs are potent surfactants, and can be effective even in low concentrations, which is useful for its therapeutic applications. Apart from excellent surfactant properties, SLs have also shown antimicrobial and antifungal activities [36–42]. It is mentioned as a cosmetic agent in the European Commission’s catalog of cosmetic ingredients as cleansing, deodorant, skin protecting and conditioning, antimicrobial, antiseborrheic, humectants, hair conditioning, and antidandruff (European Commission, 2020). It is also available commercially as a cosmetic product such as Sopholiance® S (Givaudan, 2020).

The yeast *S. bombicola* (previously known as *C. bombicola*) is a nonpathogenic yeast and it is not known to cause any disease in humans [43]. The natural habitats of *S. bombicola* are flowers and nectar-feeding insects (bumblebees). It is naturally present in honey, fermented vegetable extracts, and grape juice [43.] The yeast *S. bombicola* (previously classified as *Candida stellate*) is also present in wine fermentation and contributes to the sensory character (aroma) of wine [43,44]. Being extracellular metabolites, SLs must also be present in wines, honey, concentrated grape juice, and fermented vegetable extracts. All these facts suggest the possible biocompatibility of SLs for internal use.

## 4. Properties of SLs that make them potential agents for the treatment of COVID-19 with multiple approaches

The antiviral activities of the SLs and their derivatives have been tested against Herpes virus (Ebsteir Barr virus), human immunodeficiency virus (HIV), and influenza virus in *in-vitro* cell-free virus inactivation assay [45–48]. Viral membrane perturbation or disruption has been suggested as the possible mechanism of the virucidal activity of the SLs [48]. The acetyl groups in the structure of SL play a crucial role in promoting its antiviral activity by imparting hydrophilicity to SL [49]. The mixture of SLs has also been reported as an angiogenesis (cancer blood vessel growth) inhibitor in an *ex-vivo* rat aorta ring assay [50], while the lactonic SL (1ʹ,4ʹʹ-Sophorolactone 6ʹ,6ʹʹ-diacetate) has shown a potential anticancer agent *in-vitro* and *in-vivo* model tumor cell lines [33]. Natural SLs (mixture of acidic and lactonic SLs), as immunomodulators block the lethal effects of septic shocks in male Sprague-Dawley rats in cecal ligation and puncture model by down-regulating the cytokines and significantly improve the survival of animals when administered through intravenous and intraperitoneal injection at a dose of 5 mg/kg rat weight in 4% dimethyl sulfoxide in saline [51]. Following are the listed properties of SLs that are multitasking for the management of COVID-19.

### 4.1. Lipid solubilization

Surfactants are well known for the solubilization of the lipid membrane of bacteria and viruses. Moreover, the literature suggests that enveloped viruses are highly sensitive to the surfactants [52], and the lipid contents of the enveloped viruses are easily solubilized by the surfactants [53]. This has also been valid for the enveloped SARS-CoV-2, as WHO has recommended the use of detergents (soaps) to kill the virus on the human body surface [1]. However, the chemical surfactants/detergents are toxic and unsafe for human consumption, as they are derived from petrochemicals. The use of natural compounds such as cyclodextrins and phytosterols has been suggested to target the lipid membrane of SARS-CoV-2 [18]. Therefore, SLs could be an effective virucidal agents to kill the SARS-CoV-2 by disrupting and/or solubilizing its lipid envelope. In addition, since SLs are natural surfactants produced by nonpathogenic yeasts naturally present in wines and various food products, such as honey, we hypothesize that they are potential candidates to be used as a safe and effective preventive treatment for COVID-19.

### 4.2. Antiviral agent

SARS-CoV-2 is an RNA virus, and it is predicted that it may share functional proteins responsible for the virus replications in humans similar to other human viruses such as HIV. Therefore, antiviral drugs such as Remdesivir effective against other RNA viruses are being tested for SARS-CoV-2. Importantly, SLs has been reported to have antiviral activity against human HIV (RNA virus), Epstein-Barr virus (a Herpes DNA virus), and influenza virus (RNA virus) [45,47,48]. [48], tested different concentrations of SL derivatives against HIV and observed that SL derivatives at 3 mg/ml inactivated virus in a short period (<2 min). The acidic SL (open-ring nonacetylated SL) was found to be more virucidal than a mixture of lactonic SLs. In another *in-vitro* study, the EC_50_ (50% effective concentration) values of alkyl esters of amino acid conjugated SLs were reported to be <200 µg/ml for HIV [45]. The ethyl ester of leucine conjugated SLs (a mixture of acidic SLs) were found to be the most potent with EC_50_ value of about 25 µg/ml for HIV. Using the Epstein-Barr virus (EBV) as model organisms on Daudi lymphoid cell lines, the anti-Herpes virus activity of SL derivatives [ethyl ester di acetate SLs, ethyl ester SLs, di-acetate lactonic SLs, acidic SLs and methyl ester of SLs] was demonstrated by [46]. The ethyl ester derivative of SL (Ethyl 17-L- [(2ʹ-0-b-D-glucopyranosyl-b-D-glucopyranosyl]-oxy]-cis-9-octadecenoate), displayed the best anti-herpes activity with an EC_50_ value of <0.03 µM. The suggested dosage of tested SLs was between 2 and 30 mg/Kg body weight, via either intraperitoneal, intraarterial, or intravenous administration [46].

Interestingly, all of these viruses (HIV, influenza virus, and Epstein-Barr virus) are enveloped viruses, which further indicate that SLs could be potential antiviral agents against the enveloped SARS-CoV-2. The exact antiviral mechanism of SLs is unknown, though, it is hypothesized that it kills the virus by membrane solubilization [48]. Micelle formation around the virus and its components (genetic materials, spike proteins, etc.) could also play an important role in the antiviral properties of the SLs. The fatty acid chain length, acidic or lactonic form, and the degree of acetylation of SL can affect its antiviral activity. Shorter fatty acid chain lengths, acidic form, and diacetylate ethyl ester SLs are reported to be the most potent antiviral agents [48]. It is expected that different types of SL and its form could also have different virucidal and/or antiviral activity against SARS-CoV-2. Therefore, *in-vitro* or animal model studies for the screening of potent SLs are necessary to find out the most effective SL to kill SARS-CoV-2.

### 4.3. Immunomodulator

SARS-CoV-2 enters the host through the receptor-binding domain (RBD) present in the ‘S’ glycoprotein that interacts with ACE2 receptors in the plasma membrane of many cells, including epithelial cells of the oral mucosa [54,55]. The entry of a virus into the host cell induces cytokine storm that causes overproduction of early response pro-inflammatory cytokines like TNFα, IL-6, and IL-1β. This cytokine storm activates coagulation pathways eventually increases the risk of vascular hyperpermeability and multi-organ failure, leading to death [56]. Therefore, drugs which inhibit the immune response (particularly ‘cytokine storm’ and ‘inflammation’) to SARS-CoV-2 infection could be useful to stop or reduce the progression of the disease. Immunomodulatory drugs, for example, baricitinib and melatonin have been suggested recently for the treatment of COVID-19 [57–59]. The purified natural mixtures of SLs have immunomodulatory properties as they have been shown to down-regulate the inflammatory cytokines and up-regulate anti-inflammatory cytokines in Sprague-Dawley rats [51]. They blocks lethal effects of septic shock by significantly reducing the IL-1β (42.5%, proinflammatory cytokine), TNF-α (50%, proinflammatory cytokine), and macrophage nitric oxide (NO; 28%), and by increasing the TGF-β1 (anti-inflammatory cytokine; 11.7%) [51]. SLs have been also shown to down-regulate IL-6 in *in-vitro* cellular models using U266 (IgE producing myeloma) cells [60]. The poly(lactonic SLs) decrease (2-fold) MCP-1 (monocyte chemoattractant protein-1) in human mesenchymal stem cells [61]. Due to its immunomodulatory activity, a mixture of SLs was suggested to use for lung injury treatment [62]. Noteworthy, the expression of IL-1β, MCP-1, IL-4, IL-10, and IFN-γ is reported to be increased in COVID-19 patients. This suggests that SLs could attenuate the ‘cytokine storm’ caused by SARS-CoV-2.

### 4.4. Anticancer activity

Cancer patients have a higher risk of severe illness from COVID-19 due to their immunocompromised system. Among all cancers, patients with blood cancer have a ten-fold higher risk, and patients with metastatic cancer have about a six-fold higher risk of severe events and death [63]. SLs and their derivatives have been shown to be effective/lethal against cancer cells. For example, purified lactonic sophorolipid (1ʹ,4ʹʹ-sophorolactone 6ʹ,6ʹʹ-diacetate) effectively kills human liver cancer cells [36]. 64, reported the cytotoxicity of natural mixture of SLs and its various derivatives such as, ethyl ester, methyl ester, ethyl ester monoacetate, ethyl ester diacetate, acidic SL, and lactonic SL (1ʹ,4ʹʹ-sophorolactone 6ʹ,6ʹʹ-diacetate) against human pancreatic cancer cells [64].

65, studied the cytotoxicity of 10 purified SL molecules on human esophageal cancer cells. The authors reported that the cytotoxic effect varies with degree of saturation of fatty acid, acetylation of sophorose and lactonization or ring opening in SL. The authors concluded that the diacetylated lactonic SL with one double bond in fatty acid chain showed strongest cytotoxicity, while acidic SL showed poorest cytotoxicity against human esophageal cancer cells. The SLs and its derivatives also found effective against human cervical cancer cells [66,67]. Recently, 33, tested the antitumor activity of SL (1′,4″-Sophorolactone 6′,6″-diacetate) loaded polymeric nanocapsules using CT26 murine colon carcinoma (*in-vitro*), and female Balb/c mice (*in vivo*). After 3 days 80% inhibition in the viability of the tested cancer cells (CT26 colon cancer cells) was observed by the authors. The toxicity effect on the normal cell lines was non-significant (CCD-841-CoN normal colon epithelial cells). About 60% inhibition on the tumor growth was observed in *in-vitro* study using the encapsulated SL formulation (20–60 µM SL) [33]. The SL nanocapsules (200 µL with SL at a dose of 10 mg/kg) administered to mice, via intravenous injection inhibited the tumor growth (about 29%) and weight, without affecting the subject, which suggests that SL nanocapsules could be effectively and safely used for the treatment of colon cancer [33]. The anticancer effect of SL is due to the “depolymerization of the mitochondrial membrane and enhanced intracellular calcium levels [67]. Since anticancer effects of SL could be obtained without compromising the immune system, the SL could be a blessing for patients suffering from cancer and COVID-19. However, that needs to be tested systematically in *in-vitro* and *in-vivo* in animal systems.

## 5. The Possible mechanism of action of SL

**Figure 2.**
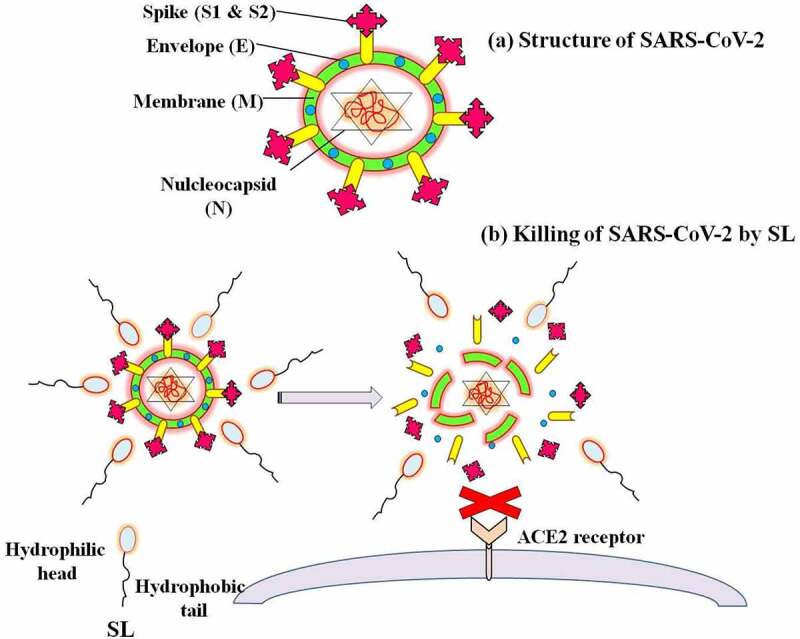
Structure of SARS-CoV-2 (a), and proposed mechanism of killing SARS-CoV-2 by sophorolipids (b)

**Figure 3.**
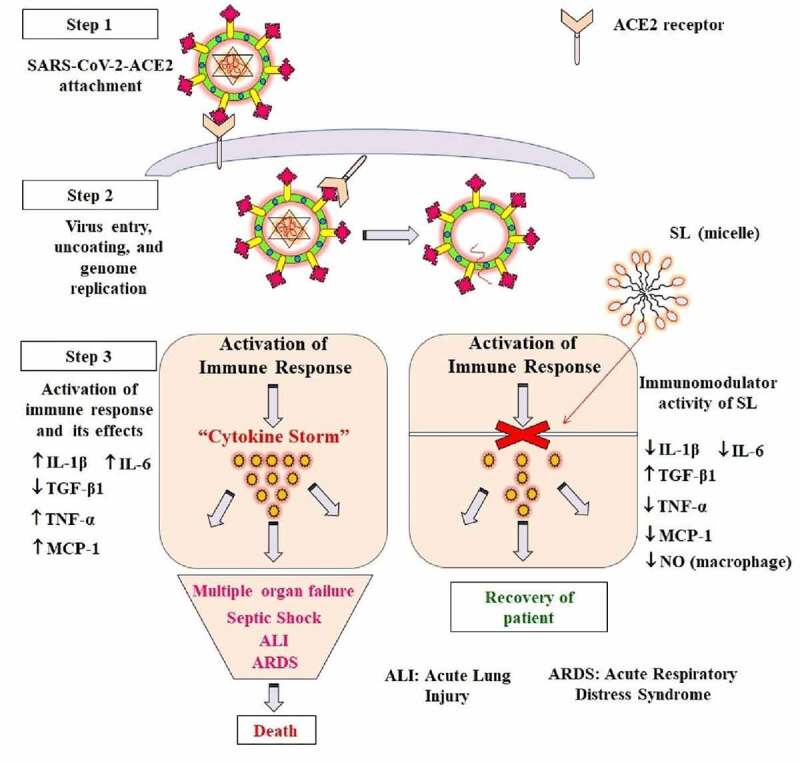
Possible mechanism of action of sophorolipids in inhibiting the progression of COVID-19 by attenuating the ‘cytokine storm’ caused by SARS-CoV-2

Like other coronaviruses, SARS-CoV-2 is a spherical or pleomorphic enveloped, positive-sense single-stranded RNA virus. The genome of coronaviruses (CoVs) encodes four major structural proteins, spike (S), envelope (E), membrane (M), and nucleocapsid (N), and several nonstructural 5 to 8 accessory proteins (Figure 2a). Among them, spike (S) glycoprotein plays an essential role in viral attachment to the ACE2 (angiotensin-Converting enzyme 2) on host cells (like a human respiratory epithelial cell) [18]. This attachment is essential for the pathogenesis of a virus. The two possible mechanisms of SL are: the solubilization of virus envelope, thus degrading the components of the virus, and inhibiting the interaction of virus and ACE2 (Figure 2b); and the inhibition of the cytokine storm and activation of anti-apoptotic genes (Figure 3). Of note, SARS-CoV-2 mutations *in-vivo* after infection may lead to the generation of several intra-host variants of the virus, one of the reasons for heterogeneous development of signs and symptoms and an unpredictable clinical outcome in infected individuals [68]. Therefore, disruption of the virus envelop could be the potential strategy to inhibit the pathogenesis of SARS-CoV-2. In addition, the administration of SL could be effective in inhibiting cytokine storms that may reduce the adverse outcome of COVID-19.

## 6. Issues and challenges associated with SLs

SLs are produced as a mixture of acidic and lactonic forms with variable lipophilic chain lengths. Due to their surfactant activity, acidic lactonic or a mixture of SLs can certainly solubilize the lipid protective outer shell of SARS-CoV-2. However, the immunomodulatory and anticancer properties of the SLs are reported in limited studies, which still need to be further verified. Particularly, the type of SLs [either acidic, lactonic, acetylated, deacetylated, or mixture] responsible for such immunomodulation and/or anticancer activities need to be verified. The crude mixture of SLs has shown varying levels of antiproliferation and anticancer activities in literature as highlighted by 69. A study reported that the purified lactonic SL [96%, C18:1] had reduced the viability of colorectal cancer, as well as normal human colonic and lung cell lines *in-vitro*, in a dose-dependent manner, while it increased the tumor burden in mice [69]. In an *in-vivo* study, the purified acidic SL (94%) decreased the cell viability of colorectal cell lines without negatively affecting the colonic epithelial and lung cell lines and highlighted the advantage of acidic SL over lactonic SL for anti-cancer activity [69]. However, *in-vivo* effects of acidic SL are not studied yet. Overall, there is not a single clinical study on the use of SLs as a therapeutic agent covering its effectiveness, side effects, and toxicity on patients. Therefore, these issues of SLs have to be addressed well before their use in any clinical studies of COVID-19 treatment.

## 7. Concluding remarks and future scope

SL is a natural surfactant that is safe for human use and is already in the market for cosmetic applications. This makes SL a perfect therapeutic candidate for the bench-to-bedside research for the treatment of COVID-19. Besides surfactant, its antiviral and immunomodulatory properties have also been established in various models (*in-vitro* cellular models and *in-vivo* animal models). It has been administered via tropical (in cosmetic products to humans), intravenous and intraperitoneal injection to animal models without showing toxicity to the tested model. So far, the major research has been focused on identifying anti-viral molecules that target the spike protein as it mediates viral entry and induces host immune responses. However, not all patients experience the same level of immune response, which could be attributed to the mutation that occurred in the genome of SARS-CoV-2. Furthermore, the presence of these biologically heterogeneous haplotypes of the virus and their variable interaction with individual genetic and epigenetic characteristics makes it difficult to predict the course of disease in a single individual. Which makes it challenging to develop universal treatment for all COVID-19 patients [70]. Therefore, a treatment like using SL-therapeutic intervention that targets SARS-CoV-2 in multiple ways could provide a better clinical outcome for COVID-19 patients than just antiviral agents alone. The Sustainable Development Goal (SDG 3: Good Health and Well-being) could also be achieved by the SL-based treatment of COVID-19.

## References

[1] World Health Organization. (2020). Coronavirus disease (COVID-19) advice for the public. https://www.who.int/emergencies/diseases/novel-coronavirus-2019/advice-for-public

[2] Gorbalenya AE, Baker SC, Baric RS, et al. The species Severe acute respiratory syndrome-related coronavirus: classifying 2019-nCoV and naming it SARS-CoV-2. Nat Microbiol. 2020;5(4):536–544. 32123347 10.1038/s41564-020-0695-z PMC7095448

[3] Wu Z, McGoogan JM. Characteristics of and important lessons from the coronavirus disease 2019 (COVID-19) outbreak in China. Jama. 2020;323(13):1239. 32091533 10.1001/jama.2020.2648

[4] Pascarella G, Strumia A, Piliego C, et al. COVID-19 diagnosis and management: a comprehensive review. J Intern Med. 2020;288(2):192–206. 32348588 10.1111/joim.13091 PMC7267177

[5] Guo, YR., Cao, QD., Hong, ZS. et al. The origin, transmission and clinical therapies on coronavirus disease 2019 (COVID-19) outbreak – an update on the status. Military Med Res 7, 11 (2020). 10.1186/s40779-020-00240-0 PMC7068984 32169119

[6] Van Doremalen N, Bushmaker T, Morris DH, et al. Aerosol and surface stability of SARS-CoV-2 as compared with SARS-CoV-1. N Engl J Med. 2020;382(16):1564–1567. 32182409 10.1056/NEJMc2004973 PMC7121658

[7] Bazant MZ, Bush JWM. A guideline to limit indoor airborne transmission of COVID-19. *Proceedings of the National Academy of Sciences of the United States of America*, 2021;118(17):e2018995118. 10.1073/pnas.2018995118 PMC8092463 33858987

[8] Greenhalgh T, Jimenez JL, Prather KA, et al. Ten scientific reasons in support of airborne transmission of SARS-CoV-2. Lancet. 2021;397(10285):1603–1605. 33865497 10.1016/S0140-6736(21)00869-2 PMC8049599

[9] Prompetchara E, Ketloy C, Palaga T. Immune responses in COVID-19 and potential vaccines: lessons learned from SARS and MERS epidemic. Asian Pac J Allergy Immunol. 2020;38(1):1–9. 32105090 10.12932/AP-200220-0772

[10] Yousefifard M, Zali A, Mohamed Ali K, et al. Antiviral therapy in management of COVID-19: a systematic review on current evidence. Arch Acad Emergency Med. 2020; 8 2: 45.http://journals.sbmu.ac.ir/aaem PMC7156260 32309809

[11] Centers for Disease Control and Prevention (CDC). Interim clinical guidance for management of patients with confirmed coronavirus disease (COVID-19) 2021; Updated on 16 February 2021. cited 2021 Aug 03. https://www.cdc.gov/coronavirus/2019-ncov/hcp/clinical-guidance-management-patients.html

[12] National Institutes of Health (NIH). USA. COVID-19 Treatment Guidelines. 2021;. cited 2021 Aug 03. https://www.covid19treatmentguidelines.nih.gov/tables/table-2e/

[13] Shakaib B, Zohra T, Ikram A, et al. A comprehensive review on clinical and mechanistic pathophysiological aspects of COVID-19 Malady: how far have we come? Virol J. 2021;18(1):1–16. 34098986 10.1186/s12985-021-01578-0 PMC8182739

[14] World Health Organization. Coronavirus disease (COVID-19): hydroxychloroquine. Dated 30 April, 2021. 2021b; cited 2021 Aug 03. https://www.who.int/news-room/q-a-detail/coronavirus-disease-(covid-19)-hydroxychloroquine

[15] World Health Organization. WHO advises that ivermectin only be used to treat COVID-19 within clinical trials. 31 March 2021.2021c; cited 2021 Aug 03. https://www.who.int/news-room/feature-stories/detail/who-advises-that-ivermectin-only-be-used-to-treat-covid-19-within-clinical-trials

[16] Li H, Zhou Y, Zhang M, et al. Updated approaches against SARS-CoV-2. Antimicrob Agents Chemother. 2020;64(6):1–7. 10.1128/AAC.00483-20 PMC7269512 32205349

[17] Rosa SGV, Santos WC. Clinical trials on drug repositioning for COVID-19 treatment. Rev Panam Salud Pública. 2020;44:1. 10.26633/RPSP.2020.40 PMC7105280 32256547

[18] Baglivo M, Baronio M, Natalini G, et al. Natural small molecules as inhibitors of coronavirus lipid-dependent attachment to host cells: a possible strategy for reducing SARS-COV-2 infectivity? Acta Biomed. 2020;91(1):161–164. 32191676 10.23750/abm.v91i1.9402 PMC7569585

[19] Daverey A, Dutta K. COVID-19: eco-friendly hand hygiene for human and environmental safety. J Environ Chem Eng. 2021;9(2):104754. 33200069 10.1016/j.jece.2020.104754 PMC7657077

[20] Jin L, Black W, Sawyer T. Application of environment-friendly rhamnolipids against transmission of enveloped viruses like sars-cov2. Viruses. 2021;13(2):2. 10.3390/v13020322 PMC7924030 33672561

[21] Smith ML, Gandolfi S, Coshall PM, et al. Biosurfactants: a Covid-19 Perspective. Front Microbiol. 2020;11(June):1–8. 32582137 10.3389/fmicb.2020.01341 PMC7295905

[22] Subramaniam MD, Venkatesan D, Iyer M, et al. Biosurfactants and anti-inflammatory activity: a potential new approach towards COVID-19. Curr Opin Environ Sci Health. 2020;17:72–81. 33015428 10.1016/j.coesh.2020.09.002 PMC7525250

[23] Ejike Ogbonna K, Victor Agu C, Okonkwo CC, et al. Use of spondias mombin fruit pulp as a substrate for biosurfactant production. Bioengineered. 2021;12(1):1–12. 33345695 10.1080/21655979.2020.1853391 PMC8806352

[24] Patel S, Homaei A, Patil S, et al. Microbial biosurfactants for oil spill remediation: pitfalls and potentials. Appl Microbiol Biotechnol. 2019;103(1):27–37. 30343430 10.1007/s00253-018-9434-2

[25] Gudiña EJ, Rangarajan V, Sen R, et al. Potential therapeutic applications of biosurfactants. Trends Pharmacol Sci. 2013;34(12):667–675. 24182625 10.1016/j.tips.2013.10.002

[26] Pacwa-Płociniczak M, Płaza GA, Piotrowska-Seget Z, et al. Environmental applications of biosurfactants: recent advances. Int J Mol Sci. 2011;12(1):633–654. 21340005 10.3390/ijms12010633 PMC3039971

[27] Shah V, Daverey A. Effects of sophorolipids augmentation on the plant growth and phytoremediation of heavy metal contaminated soil. J Clean Prod. 2021;280:124406.

[28] Huang X, Lu Z, Zhao H, et al. Antiviral activity of antimicrobial lipopeptide from Bacillus subtilis fmbj against pseudorabies virus, porcine parvovirus, newcastle disease virus and infectious bursal disease virus in vitro. Int J Pept Res Ther. 2006;12(4):373–377.

[29] Kang BR, Park JS, Jung WJ. Antiviral activity by lecithin-induced fengycin lipopeptides as a potent key substrate against Cucumber mosaic virus. Microb Pathog. 2021;155(December2020):104910. 33930417 10.1016/j.micpath.2021.104910

[30] Huang C, Hu C, Sun G, et al. Antimicrobial finish of cotton fabrics treated by sophorolipids combined with 1,2,3,4-butanetetracarboxyic acid. Cellulose. 2020;27(5):2859–2872.

[31] Daverey A, Pakshirajan K. Sophorolipids from Candida bombicola using mixed hydrophilic substrates: production, purification and characterization. Colloids Surf B Biointerfaces. 2010;79(1):246–253. 20427162 10.1016/j.colsurfb.2010.04.002

[32] Elshafie AE, Joshi SJ, Al-Wahaibi YM, et al. Sophorolipids production by candida bombicola ATCC 22214 and its potential application in microbial enhanced oil recovery. Front Microbiol. 2015;6(3):743–753. 26635782 10.3389/fmicb.2015.01324 PMC4659913

[33] Haggag Y, Elshikh M, El-Tanani M, et al. Nanoencapsulation of sophorolipids in PEGylated poly(lactide-co-glycolide) as a novel approach to target colon carcinoma in the murine model. Drug Deliv Transl Res. 2020;10(5):1353–1366. 32239473 10.1007/s13346-020-00750-3 PMC7447623

[34] Ribeiro IA, Bronze MR, Castro MF, et al. Design of selective production of sophorolipids by *Rhodotorula bogoriensis* through nutritional requirements. J Mol Recog. 2012;25(11):630–640. 10.1002/jmr.2188 23108623

[35] Jiménez-Peñalver P, Rodríguez A, Daverey A, et al. Use of wastes for sophorolipids production as a transition to circular economy: state of the art and perspectives. Rev Environ Sci Biotechnol. 2019;18(3):413–435.

[36] Chen J, Song X, Zhang H, et al. Sophorolipid produced from the new yeast strain Wickerhamiella domercqiae induces apoptosis in H7402 human liver cancer cells. Appl Microbiol Biotechnol. 2006;72(1):52–59. 16528516 10.1007/s00253-005-0243-z

[37] Gaur VK, Regar RK, Dhiman N, et al. Biosynthesis and characterization of sophorolipid biosurfactant by Candida spp.: application as food emulsifier and antibacterial agent. Bioresour Technol. 2019;285(April):121314. 30992159 10.1016/j.biortech.2019.121314

[38] Haque F, Verma NK, Alfatah M, et al. Sophorolipid exhibits antifungal activity by ROS mediated endoplasmic reticulum stress and mitochondrial dysfunction pathways in: Candida albicans. RSC Adv. 2019;9(71):41639–41648. 10.1039/c9ra07599b PMC9076456 35541620

[39] Mendes RM, Francisco AP, Carvalho FA, et al. Fighting S. aureus catheter-related infections with sophorolipids: electing an antiadhesive strategy or a release one? Colloids Surf B Biointerfaces. 2021;208:112057. 34464911 10.1016/j.colsurfb.2021.112057

[40] Sen S, Borah SN, Kandimalla R, et al. Sophorolipid biosurfactant can control cutaneous dermatophytosis caused by trichophyton mentagrophytes. Front Microbiol. 2020;11(March):1–15. 32226417 10.3389/fmicb.2020.00329 PMC7080852

[41] Silveira VAI, Nishio EK, Freitas CAUQ, et al. Production and antimicrobial activity of sophorolipid against Clostridium perfringens and Campylobacter jejuni and their additive interaction with lactic acid. Biocatal Agric Biotechnol. 2019;21(August):101287.

[42] Tang Y, Ma Q, Du Y, et al. Efficient purification of sophorolipids via chemical modifications coupled with extractions and their potential applications as antibacterial agents. Sep Purif Technol. 2020;245(April):116897.

[43] De Graeve M, De Maeseneire SL, Roelants SL, et al. Starmerella bombicola, an industrially relevant, yet fundamentally underexplored yeast. FEMS Yeast Res. 2018;18(7):foy072. 10.1093/femsyr/foy072 29982357

[44] Šipiczki M, Ciani M, Csoma H. Taxonomic reclassification of *Candida stellata* DBVPG 3827. Folia Microbiol. 2005;50:494–498. 16681146 10.1007/BF02931436

[45] Azim A, Shah V, Doncel GF, et al. Amino acid conjugated sophorolipids: a new family of biologically active functionalized glycolipids. Bioconjug Chem. 2006;17(6):1523–1529. 17105232 10.1021/bc060094n

[46] Gross RA, Shah V. 2007. Anti-herpes virus properties of various forms of sophorolipids. In *World patent 2007130738 A1*.

[47] Gross RA, Shah V, Doncel GF. 2004. *United States Patent Application Publication Pub. No .: US 2004/0121015 A1*.

[48] Shah V, Doncel GF, Seyoum T, et al. Sophorolipids, microbial glycolipids with anti-human immunodeficiency virus and sperm-immobilizing activities. Antimicrob Agents Chemother. 2005;49(10):4093–4100. 16189085 10.1128/AAC.49.10.4093-4100.2005 PMC1251511

[49] Borsanyiova M, Patil A, Mukherji R, et al. Biological activity of sophorolipids and their possible use as antiviral agents. Folia Microbiol (Praha). 2016;61(1):85–89. 26126789 10.1007/s12223-015-0413-z

[50] Mohamed SK, Asif M, Nazari MV, et al. Antiangiogenic activity of sophorolipids extracted from refined bleached deodorized palm olein. Indian J Pharmacol. 2018;49(5):344–347. 10.4103/ijp.IJP_312_18 PMC6444841 31031467

[51] Bluth MH, Kandil E, Mueller CM, et al. Sophorolipids block lethal effects of septic shock in rats in a cecal ligation and puncture model of experimental sepsis. Crit Care Med. 2006;34(1):E188. 10.1097/01.ccm.0000196212.56885.50 16374148

[52] Strauss JH, Strauss EG. The structure of viruses. Viruses and human disease. Vol. 80. Massachusetts, USA, Elsevier; 2008. p. 35–62.

[53] Conley L, Tao Y, Henry A, et al. Evaluation of eco-friendly zwitterionic detergents for enveloped virus inactivation. Biotechnol Bioeng. 2017;114(4):813–820. 27800626 10.1002/bit.26209

[54] Hoffmann M, Kleine-Weber H, Schroeder S, et al. SARS-CoV-2 cell entry depends on ACE2 and TMPRSS2 and is blocked by a clinically proven protease inhibitor. Cell. 2020;181(2):271–280.e8. 32142651 10.1016/j.cell.2020.02.052 PMC7102627

[55] Xu H, Zhong L, Deng J, et al. High expression of ACE2 receptor of 2019-nCoV on the epithelial cells of oral mucosa. Int J Oral Sci. 2020;12(1):8. 32094336 10.1038/s41368-020-0074-x PMC7039956

[56] Jose RJ, Manuel A. COVID-19 cytokine storm: the interplay between inflammation and coagulation. Lancet Respir Med. 2020;8(6):e46–e47. 32353251 10.1016/S2213-2600(20)30216-2 PMC7185942

[57] Richardson P, Griffin I, Tucker C, et al. Baricitinib as potential treatment for 2019-nCoV acute respiratory disease. Lancet. 2020;395(10223):e30–e31. 32032529 10.1016/S0140-6736(20)30304-4 PMC7137985

[58] Stebbing J, Phelan A, Griffin I, et al. COVID-19: combining antiviral and anti-inflammatory treatments. Lancet Infect Dis. 2020;20(4):400–402. 32113509 10.1016/S1473-3099(20)30132-8 PMC7158903

[59] Zhang R, Wang X, Ni L, et al. COVID-19: melatonin as a potential adjuvant treatment. Life Sci. 2020;250(February):117583. 32217117 10.1016/j.lfs.2020.117583 PMC7102583

[60] Hagler M, Smith-Norowitz TA, Chice S, et al. Sophorolipids Decrease IgE Production in U266 Cells by Downregulation of BSAP (Pax5), TLR-2, STAT3 and IL-6. J Allergy Clin Immunol. 2007;119(1):S263.

[61] Arabiyat AS, Diaz-Rodriguez P, Erndt-Marino JD, et al. Effect of poly(sophorolipid) functionalization on human mesenchymal stem cell osteogenesis and immunomodulation. ACS Appl Bio Mater. 2019;2(1):118–126. 10.1021/acsabm.8b00434 35016334

[62] Wadgaonkar R, Gross RA, Butnariu D, et al. *Lung Injury Treatment Pct/US08/62759*; 2010. https://patentimages.storage.googleapis.com/7d/2a/15/6473be8dbface8/US20100130442A1.pdf

[63] Liang W, Guan W, Chen R, et al. Cancer patients in SARS-CoV-2 infection: a nationwide analysis in China. Lancet Oncol. 2020;21(3):335–337. 32066541 10.1016/S1470-2045(20)30096-6 PMC7159000

[64] Fu SL, Wallner SR, Bowne WB, et al. Sophorolipids and their derivatives are lethal against human pancreatic cancer cells. J Surg Res. 2008;148(1):77–82. 18570934 10.1016/j.jss.2008.03.005

[65] Shao L, Song X, Ma X, et al. Bioactivities of sophorolipid with different structures against human esophageal cancer cells. J Surg Res. 2012;173(2):286–291. 21035135 10.1016/j.jss.2010.09.013

[66] Li H, Guo W, Ma X, et al. In vitro and in vivo anticancer activity of sophorolipids to human cervical cancer. Appl Biochem Biotechnol. 2017;181(4):1372–1387. 27796874 10.1007/s12010-016-2290-6

[67] Nawale L, Dubey P, Chaudhari B, et al. Anti-proliferative effect of novel primary cetyl alcohol derived Sophorolipids against human cervical cancer cells HeLa. PLoS ONE. 2017;12(4):1–14. 10.1371/journal.pone.0174241 PMC5395175 28419101

[68] Shen Z., Xiao Y., Kang L., et al. Genomic Diversity of Severe Acute Respiratory Syndrome-Coronavirus 2 in Patients with Coronavirus Disease 2019. Clin Infect Dis. 2020;71(15):713–720. 32129843 10.1093/cid/ciaa203 PMC7108196

[69] Callaghan B, Lydon H, Roelants SLKW, et al. Lactonic sophorolipids increase tumor burden in apcmin± mice. PLOS ONE. 2016;11(6):e0156845. 27271048 10.1371/journal.pone.0156845 PMC4894592

[70] Lippi G, Sanchis-Gomar F, Henry BM. Coronavirus disease 2019 (COVID-19): the portrait of a perfect storm. Ann Transl Med. 2020;8(7):497. 32395541 10.21037/atm.2020.03.157 PMC7210187

[71] Inès M, Dhouha G. Glycolipid biosurfactants: potential related biomedical and biotechnological applications. Carbohydr Res. 2015;416:59–69. 26359535 10.1016/j.carres.2015.07.016

[72] Haferburg D, Hommel R, Kleber H‐P, et al. Antiphytovirale Aktivität von Rhamnolipid aus Pseudomonas aeruginosa. Acta Biotechnologica. 1987;7(4):353–356.

